# One-step methodology for the direct covalent capture of GPCRs from complex matrices onto solid surfaces based on the bioorthogonal reaction between haloalkane dehalogenase and chloroalkanes†
†Electronic supplementary information (ESI) available. See DOI: 10.1039/c7sc03887a


**DOI:** 10.1039/c7sc03887a

**Published:** 2017-10-19

**Authors:** Kaizhu Zeng, Qian Li, Jing Wang, Guowei Yin, Yajun Zhang, Chaoni Xiao, Taiping Fan, Xinfeng Zhao, Xiaohui Zheng

**Affiliations:** a Key Laboratory of Resource Biology and Biotechnology in Western China , Ministry of Education , College of Life Sciences , Northwest University , Xi’an 710069 , China . Email: zhaoxf@nwu.edu.cn ; Fax: +86 029 88302686 ; Tel: +86 029 88302686; b Department of Biochemistry and Biophysics , University of North Carolina at Chapel Hill , NC , USA; c Department of Pharmacology , University of Cambridge , Cambridge CB2 1PD , UK

## Abstract

Protein immobilization techniques play an important role in the development of assays for disease diagnosis and drug discovery. However, many of these approaches are not applicable to transmembrane proteins. G protein-coupled receptors (GPCRs) are the largest protein superfamily encoded by the human genome and are targeted by a quarter of all prescription drugs. GPCRs are highly dynamic and sensitive to changes in the ambient environment, and current immobilization methodologies are not suitable for GPCRs. We used haloalkane dehalogenase (Halo) as an immobilization tag fused to the β_2_-adrenoceptor (β_2_-AR), angiotensin II type 1 (AT_1_) and angiotensin II type 2 (AT_2_) receptors. The engineered Halo-tag covalently binds to a specific substrate chloroalkane through Asp 106 in the catalytic pocket. The Halo-tagged GPCRs were expressed in *Escherichia coli* at a suitable yield. Accordingly, we loaded cell lysate containing Halo-tagged GPCRs onto a macroporous silica gel coated with chloroalkane. Morphological characterization indicated a homogeneous monolayer of immobilized Halo-tagged GPCRs on the silica gel surface. The immobilized receptors proved to be surrounded by specific bound phospholipids including PG C18:1/C18:1. We observed a radio-ligand binding ability and ligand-induced conformational changes in the immobilized GPCRs, suggesting the preservation of bioactivity. This method is a one-step approach for the specific immobilization of GPCRs from cell lysates and validates that immobilized receptors retain canonical ligand binding capacity. Our immobilization strategy circumvents labor-intensive purification procedures and minimizes loss of activity. The immobilized receptors can be applied to high-throughput drug and interaction partner screening for GPCRs.

## Abstract 2

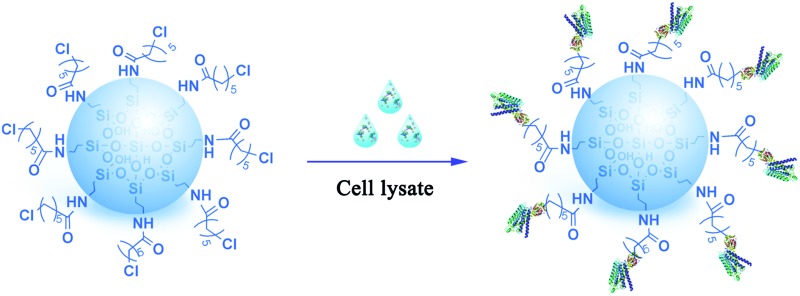
An approach is established for the specific immobilization of GPCRs from cell lysates that circumvents labor intensive purification procedures and minimize loss of activity.

## Introduction

Anchoring protein-based techniques^1^–^5^ are central to biomarker detection, proteomic screens, drug discovery and the systemic study of biological mechanisms at the molecular level.^6^ These methods require the immobilization of target proteins onto solid scaffolds. As such, one challenge for these techniques is the conservation of target protein bioactivity during immobilization, and this obstacle is of particular concern with regard to transmembrane proteins.^7^,^8^

Nonspecific methodologies, including physical adsorption and covalent methods, are commonly utilized for protein immobilization. These methods can lead to protein denaturation and heterogeneous orientations of immobilized proteins. As an alternative, site-specific immobilization is an optimal approach used to fabricate homogeneous, reproducible and controllable substrate-protein surfaces.^9^ Site specificity can be achieved with the noncovalent recognition of biomolecules.^10^–^13^ This method is challenged by linkage stability and the stability of immobilized protein. Furthermore, covalent site-specific immobilization is superior for minimizing protein dissociation. Based on ongoing developments in click chemistry, a number of protein immobilization methods have been developed.^14^–^17^ These methodologies rely on endogenous amino acids that might lead to undesired proteins bound to the surface. Impressive achievements in protein engineering have led to the use of O^6^-alkylguanine-DNA alkyltransferase and farnesyltransferase as tags to immobilize target proteins on surfaces.^18^,^19^ This strategy has mainly been applied to cytosolic proteins with good solubility in the aqueous phase but rarely to transmembrane proteins with important biological functions such as G protein-coupled receptors (GPCRs).

GPCRs are associated with almost every major therapeutic category, including pain, asthma, inflammation, obesity, and cancer as well as cardiovascular, metabolic, gastrointestinal and central nervous system diseases. They represent the single most important class of drug targets – ∼50% of current drugs target GPCRs, and ∼20% of the top 50 best-selling drugs target GPCRs.^20^ The dominance of GPCRs as a drug target class highlights the need for functional molecular assays to assess activation and screening for specific ligands,^21^ which can be addressed with a strategy based on GPCR immobilization. However, GPCR immobilization is challenging, as the receptors are sensitive to changes in the chemical environment. Thus, improved covalent, site-specific immobilization techniques are urgently needed.

In this paper, we engineered haloalkane dehalogenase (Halo) as a tag on the C-terminus of three GPCRs: β_2_-adrenoceptor (β_2_-AR), angiotensin II type 1 (AT_1_) and angiotensin II type 2 (AT_2_). We modified a macroporous silica gel with 6-chlorohexanoic acid derivatives that are Halo-tag substrates. We captured the three Halo-tagged receptors from an *Escherichia coli* lysate onto the gel surface. The immobilized receptors exhibited ligand-binding activities and ligand-induced conformational changes. This study reports for the first time that bioorthogonal chemical reactions between enzymes and substrates have been applied to immobilized β_2_-AR, AT_1_ and AT_2_ receptors. Our approach is fast, robust, high-yielding, chemoselective and minimizes GPCR activity loss.

## Results and discussion

### *E. coli* expression of Halo-tagged receptors

**Fig. 1:**
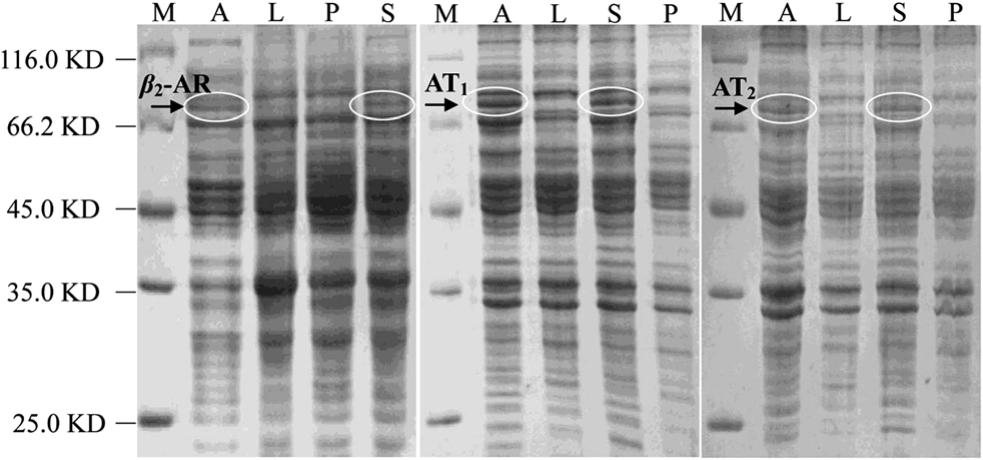
SDS-PAGE analysis of cell lysates from *E. coli* expressing Halo-tagged β_2_-AR, AT_1_ or AT_2_. Lane M: protein marker; Lane A: auto-induction growth medium; Lane L: LB medium; Lane S: supernatant; Lane P: precipitation.

As Halo-tags form covalent intermediates by removing halides from aliphatic hydrocarbons,^22^,^23^ they have been successfully applied in the expression and purification of soluble proteins and cell surface labelling.^24^–^27^ In *E. coli*, we expressed β_2_-AR, AT_1_ and AT_2_ receptors fused with a Halo-tag at their C-terminus after auto-induction. The cell lysates were directly analysed by SDS-PAGE (Fig. 1). In the marker lane, a linear relationship was established between mobility and the logarithm of the molecular weight, which was used to calculate the molecular weight of the experimental bands. Compared with the LB media lanes, a sharp new band was visible in the auto-induction media. Further analysis of cells incubated in these media showed that a dominant band exhibiting mobility consistent with the expected protein appeared in the supernatant of the cell lysates. The molecular weights of the three Halo-tagged receptors were determined to be 80.0, 74.5 and 74.8 kDa. Importantly, it demonstrates that milligram quantities of functional, stable GPCRs can be expressed, a challenging task for high-resolution structural studies and other research.

### Selection of a halide linker for halo-receptor immobilization

As a powerful anchor, the Halo-tag has been successfully applied in the development of diverse assays, revealing a complexity inherent to cellular processes.^28^–^32^ The length and structure of the linker are critical for assay stability.^33^ We designed five linkers ending with chlorine groups (ESI Table 1†) and tested their reactivity with Halo-tagged receptors. Using the Halo-tag® TMR ligand as a marker ligand, the reactivity was determined from the fluorescence signal of residual receptors that were not immediately immobilized after the reaction. Our results indicated that the Halo-tagged receptors covalently bind to all five linkers to release the halide, but linker 3 exhibited the best reactivity. To evaluate the chemical properties responsible for the reactivity difference, we calculated the partition coefficient (*X* log *P*) values, an index of hydrophobicity. Linker reactivity was correlated to the *X* log *P* value when the total atom number of the spacer arm was less than 13. Linker 3 demonstrated much higher reactivity than linker 2 although their spacer arms contained the same number of carbons. This might ascribe the presence of a large benzene ring in linker 2 that prevents the chloroalkane from reaching the Halo-tag catalytic site. Linker 4 contains one more carbon atom than linker 3 before the chloride, resulting in a higher *X* log *P* value of 1.2. It demonstrated lower reactivity partly because the total atom number of the spacer between the enzyme and the silica gel surface was too long. These results indicate that bulky substrates with higher hydrophobicity are less likely to form an ester bond with the fused Halo-tag and provide a strategy to design new linkers to ensure the efficient immobilization of Halo-tagged proteins. In summary, we expressed Halo-tagged GPCRs and demonstrated that our approach can be used to covalently capture transmembrane receptors from the cell lysate mixture efficiently in one step.

Because the receptors in the *E. coli* lysates utilize the Halo-tag to react specifically with a linker-derivatized silica gel, this methodology can minimize the nonspecific attachment of proteins. The nonspecific immobilization of proteins by traditional chemical means results in significant activity loss, making the method described herein particularly suitable for quantitative GPCR immobilization with negligible activity loss. Using previous techniques, ionic detergent micelles and lipid-detergent micelles were used to dissociate receptors from their natural environment.^34^ However, detergents often cause receptor misfolding and degradation. In contrast, this methodology combines the use of lysozymes and low-frequency sonication for optimal receptor extraction.

### Surface characterization

The search for a universally applicable protocol to characterize protein-coated supports continues due to the diversity of materials used to immobilize proteins. A wide range of analytical methods has been employed to solve this problem, including elemental analysis, thermal analysis and electron microscopy. Although such analyses can provide physical and chemical information, the functionality of the immobilized proteins remains unknown. We used laser scanning confocal microscopy (LSM) to confirm normal receptor function in receptor-conjugated silica gel microspheres in addition to physical characterization *via* X-ray photoelectron spectroscopy (XPS), cryo-field emission scanning electron microscopy (SEM) and high-resolution transmission electron microscopy (TEM).

### Composite characterization

The surface features of the linker- and receptor-conjugated microspheres were first characterized by XPS. A survey scan of the bare silica gel provided specific signals for O 1s, Si 2s and Si 2p with a molar ratio of 1 : 2 (O : Si), indicating satisfactory gel purity, as the surface of the silica microspheres is reported to be either siloxane (Si–O–Si) or silanol (Si–OH).^33^ Thus, it was reasonable to assume that the C 1s peak from the immobilized receptors comes from the linker and protein layer. To verify this assumption, we assessed the XPS character of the control supports (aminopropyl gel and 6-chlorohexanoic acid coated gel) and the three immobilized receptors. Compared with the bare silica gel, the aminopropyl gel and 6-chlorohexanoic acid-coated gel exhibited appreciable C 1s signals with a carbon content of 0.97 and 1.06 atom%, respectively, indicating successful modification of the silica by the linkers.

The O 1s spectra for the receptor-conjugated microspheres showed a broad featureless peak of 1.4 eV full width at half-maximum (FWHM) at 532.1 eV with a high BE shoulder. N 1s was also featureless, with peaks at 403 eV. These results are similar to the characteristics of free peptides.^35^ Thus, the C 1s peak was the most promising signal for XPS characterization of the three receptors. The C 1s signal from the silica-linker-modified receptors exhibited six different signals at 284.92, 286.14, 286.93, 288.30, 288.94 and 289.23 eV (ESI Table 2†), which were fitted by Gaussian–Lorentzian functions using a Shirley-type background. The peaks were attributed to carbon atoms with C–C (284.92 eV), C–N (286.14 eV), C–O (286.93 eV), O=C–N (288.30 eV), O=C–OH (288.94 eV) and C–N_3_ (289.23 eV) configurations. The peak positions agree with previously reported binding energies for low molecular weight organic compounds.^36^,^37^ The chemical shift intensities at these six positions were proportional to the population of the particular carbon type, and these chemical shifts were consistent with the six potential chemical environments of the carbon atoms in the proteins. There was little difference between the C 1s shape for free or immobilized receptors because the number of carbon atoms in the linker was at least 1000 times less than that in the free receptors. All of the three immobilized receptors exhibited much higher carbon content (∼59.6 atom%) than the control supports, indicating that these receptors were successfully conjugated onto the silica gel microspheres.

### Morphological analysis

**Fig. 2:**
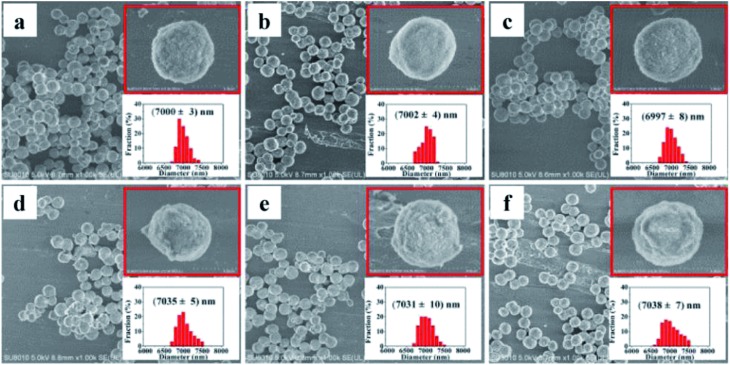
SEM micrographs of silica gel coatings: (a) bare silica gel; (b) aminopropyl silica gel; (c) 6-chlorohexanoic acid-coated gel; (d) β_2_-AR-coated gel; (e) AT_1_-coated gel; (f) AT_2_-coated gel.

SEM images of the bare silica gel exhibited a spherical morphology with a relatively uniform and porous structure and a diameter of 7.0 ± 0.003 μm (Fig. 2a). The morphology and size distribution did not show obvious differences after reaction with γ-aminopropyltriethoxysilane followed by 6-chlorohexanoic acid (Fig. 2b and c); the linkers had little effect on the silica gel structure. Larger particles with a diameter of 7.035 ± 0.005 μm were observed when 6-chlorohexanoic-silica was treated with β_2_-AR, AT_1_ and AT_2_ (Fig. 2d–f). The changes were confirmed at higher magnifications (ESI Fig. 1†). GPCRs such as β_2_-AR, AT_1_ and AT_2_ have diameters of approximately 10.0 nm.^38^,^39^ Taking the size of the Halo-tag into account, the diameters of the three Halo-tagged receptors were calculated to be approximately 20.0 nm, which is ∼50% of the change in size of the receptor-conjugated microspheres compared to the control supports. This result confirmed that the surfaces of the microspheres were evenly coated with a monolayer of the receptors.

As shown in the insets of Fig. 2, distinct features of the receptor-conjugated microspheres were observed at higher magnification. All of the three receptor-conjugated microspheres exhibited a rough surface compared to the bare microspheres. To examine if the surface appearance was caused by the Halo-tagged receptor coatings, the receptor-conjugated microspheres and bare microspheres were calcinated at 600 °C for 6.0 h. After destroying the receptors at high temperature, the receptor-conjugated microspheres were similar in size and morphology to the bare microspheres, as expected (ESI Fig. 2†). This observation suggested that the morphological changes observed in the receptor-conjugated microspheres were attributable to the immobilized receptors.

Subsequent experiments using TEM clearly demonstrated that receptors were bound to the entire surface of the microspheres. Distinct irregular protuberances were visualized on the receptor-conjugated microspheres (ESI Fig. 3†). Elemental analysis of the protuberances revealed high carbon content, confirming that receptor immobilization had occurred. The receptor-conjugated microspheres appeared as a milky and gleaming layer over the spherical surface, indicating the presence of a homogeneous layer of receptors; no such layer was observed on the control supports. Immuno-TEM images of the β_2_-AR-conjugated microspheres displayed a larger size and a rougher surface than the 6-chlorohexanoic acid coated gel, thus confirming this layer (ESI Fig. 4†).

**Fig. 3:**
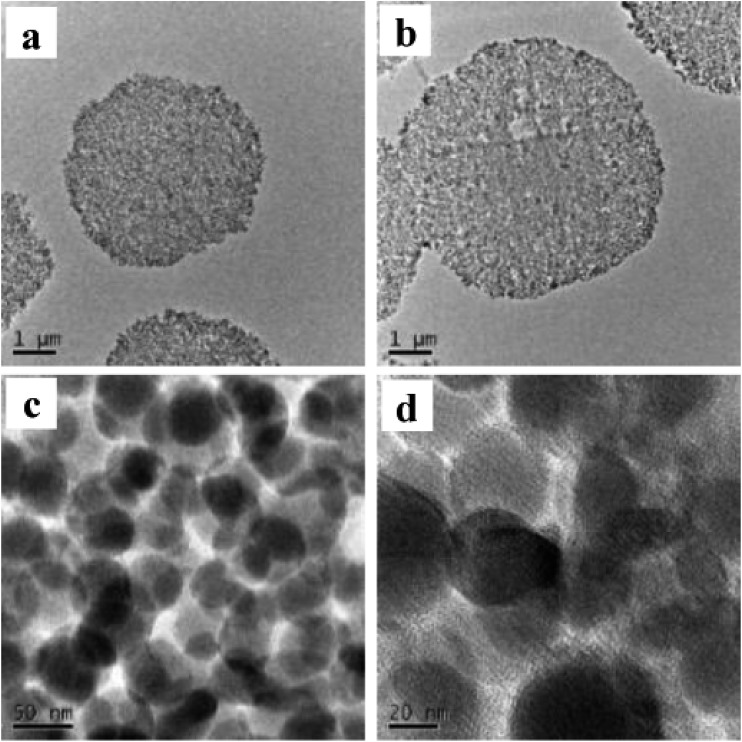
TEM images of a 40 nm cross-section of linker-coated silica gel at magnification of 2500× (a) and receptor-conjugated silica gel at magnifications of 2500× (b), 63 000× (c) and 145 000× (d).

**Fig. 4:**
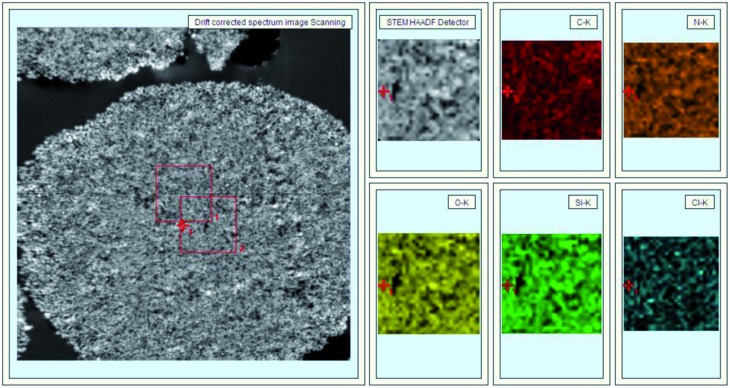
STEM-HAADF image (left) and STEM-EDS chemical maps (right) of a β_2_-AR-conjugated silica gel cross-section.

In Fig. 3, 40 nm cross-sections of linker-coated and receptor-conjugated microspheres imaged with TEM clearly show microspheres with dense and sharp differences from one another. The pores within the microsphere are visible as light areas, whereas the matrices of the silica gel can be seen as dark regions. The continuous grey layer identified as an epoxy resin was clearly discriminated from the silica gel. The control supports and immobilized receptors exhibited irregularly shaped pores with a large distribution, which is in agreement with previous reports of wide-pore silica gels.^40^ At higher magnifications (Fig. 3), the dark matrices were surrounded by a thin grey film, where we speculated the Halo-tagged receptors are located. When the cross-section was placed over a Quantifoil/Cu hole to minimize background interference, larger areas of grey film were observed in the receptor-conjugated microspheres, and their pores appeared more filled compared to the linker-coated supports. This result was further confirmed by high angle annular dark field imaging (HAADF) and dual energy dispersive spectroscopy (EDS). At the end of each line of STEM-EDS acquisition, quick HAADF images were obtained to check and eliminate any residual sample-drift by cross-correlation. The image was acquired at 256 × 256 pixels with multiple frame acquisition, with a total acquisition time of approximately 10 min. The STEM-HAADF image and STEM-EDS chemical maps for the β_2_-AR conjugated microsphere cross-section were shown in Fig. 4. Compared to the images of the control spheres (ESI Fig. 5†), Fig. 4 demonstrated the non-homogeneous and spatial elemental distribution of carbon, oxygen and nitrogen across the spheres. It is evident that the three receptor-conjugated spheres are richer in these elements than the control spheres. This finding was also confirmed with EDS (ESI Fig. 6†); the weight percentages of carbon, oxygen and nitrogen in the receptor-conjugated spheres were substantially higher than in the control sphere. These results indicate that the linker-modified silica gel was successfully coated with Halo-tagged receptors. Collectively, from the XPS, SEM and TEM data, we concluded that all three Halo-tagged receptors are immobilized on the silica gel as a homogenous monolayer.

### Function of the immobilized receptors

#### The immobilized receptors bound preferentially to specific phospholipids

Phospholipids are key players in modulating the structure and function of membrane proteins. Their effects on GPCRs have been studied by following protein function after reconstitution in given environments. To investigate whether the immobilized receptors are surrounded by certain bound lipids after immobilization, we extracted the receptor-conjugated silica gel by the method of Bligh and Dyer.^41^ We then identified and quantitatively measured the phospholipids in the extraction. This allowed us to compare the relative amounts of certain phospholipids bound to the immobilized receptor with those found in *E. coli* membranes.

The three immobilized receptors appeared to favor the binding of specific PG species such as PG C18:1/C18:1 (ESI Fig. 7 and Table 3†). The total specific PG species found on each immobilized receptor was increased up to 8-fold compared with their abundance in the bacterial cell membrane. For the case of PE, we observed lower relative enrichment. These results were anticipated since the use of detergent for receptor solubilization was avoided in this work. Taking inspiration from previous reports where the modulation of lipids on membrane protein structure and activity are attributed to the binding of specific lipids to the protein surface even in the absence of a bilayer,^42^ we reasoned that the functions of the three immobilized receptors were partly owing to their surrounding environments of bound lipids.

#### The immobilized receptors exhibited radio-ligand binding ability

Saturation experiments with [^125^I]-CYP as a radio-ligand showed specific saturated binding isotherms. The high-affinity binding data of CYP to both the cells and the immobilized β_2_-AR were well fitted to a one-site hyperbolic binding curve using nonlinear regression analysis (ESI Fig. 8a†). The total number of receptors (*B*_max_) on the desired cells and the receptor-conjugated microsphere were 330 ± 6 pmol per mg protein and 423 ± 7 nmol per g gel. The dissociation constants were measured as 19.34 ± 1.04 pM (*n* = 10) and 19.90 ± 0.91 pM (*n* = 10). The specific β_2_-AR antagonist (ICI 118,551) displaced CYP in a monophasic displacement pattern, resulting in an IC_50_ value of 14.5 ± 1.1 nmol l^–1^ for the immobilized receptor (ESI Fig. 8b†). [^125^I]-[Sar^1^, Ile^8^]AngII displayed a higher affinity for AT_1_ than AT_2_ not only in *E. coli* cells but also on the surface of the silica gel. The dissociation constants of the interactions between [^125^I]-[Sar^1^, Ile^8^]AngII and the two immobilized receptors were 0.28 ± 0.02 and 0.087 ± 0.006 nM (ESI Fig. 9a†), respectively. Competitive binding analysis exhibited IC_50_ values of 4.80 ± 0.04 nmol l^–1^ for losartan to AT_1_ and 0.84 ± 0.05 nmol l^–1^ for PD123319 to immobilized AT_2_ (ESI Fig. 9b†). These data demonstrate that there is a high population of β_2_-AR, AT_1_ or AT_2_ on the three kinds of silica gel. The immobilized receptors were specific in recognizing their ligands, and thereby had ligand induced pharmacological functions.

#### The immobilized receptors demonstrated ligand-induced conformational changes

We applied LSM to gain further insight into the activity, orientation and conformation of the immobilized receptors. All of the receptors in this work were created using a synthetic gene to encode the particular receptor with the desired C-terminal enzyme tag. This structural property requires the receptor domain to specifically recognize its ligand and the tag to efficiently bind to an optimal linker. To ensure the functional integrity of the three Halo-tagged receptors, we measured the activities of their enzyme domains and ligand-binding regions.

First, we examined the activity of dehalogenase incorporated in the receptors, which were expressed in *E. coli*. A fluorescent carboxytetramethylrhodamine ligand (Halo-tag® TMR ligand) was utilized to recognize and label the dehalogenase domains of the fusion receptors for imaging analysis using LSM. Compared with a control vector not expressing the receptors, clear fluorescence signals were observed in the *E. coli* expressing β_2_-AR, AT_1_ and AT_2_ (ESI Fig. 10†), indicating that dehalogenase was successfully incorporated into the structures of the three receptors. This result also confirmed that the dehalogenase in the receptors could form a covalent ester bond with haloalkane functionalities *via* the nucleophilic displacement of halides. The next set of experiments was designed to reveal whether the dehalogenase fused to the receptors retained catalytic activity in a solid–liquid interface. To this end, we covalently linked salbutamol (a specific ligand for β_2_-AR) and angiotensin II (a specific ligand for AT_1_ and AT_2_) onto silica gel (ESI Fig. 11†) to prepare affinity adsorbents capable of capturing the target receptors and orienting the dehalogenase domain outwards. We stated that the attachment of salbutamol may affect its binding to β_2_-AR. Such an influence was ignorable when investigating the catalytic activity of the three Halo-tagged receptors using the current strategy. As a non-catechol strong partial agonist of β_2_-AR, salbutamol has a protonated secondary amine nitrogen with a tertiary butyl group attached to it, a β-OH group, one OH group and another –CH_2_OH group on the aromatic ring. These functional groups produce several kinds of binding site to β_2_-AR. Asp^113^ makes a strong salt-bridge contact with the protonated amine (distance, 2.9 Å). Due to the long –CH_2_OH group of salbutamol, a smaller rotation of TM5 places the serines in close proximity to the hydroxyl groups of the drug. Asp^113^ simultaneously generates a hydrogen bond with the β-OH. The presence of a bulky alkyl –(CH_3_)_3_ group at one end enables salbutamol to interact with TM7. Leu^311^ shows favorable van der Waals interaction with the alkyl group of salbutamol. In this work, salbutamol was attached on the surface of silica gel through the use of phenol hydroxyl. There is a linker of six atoms between the gel surface and salbutamol, which allows salbutamol to be flexible and rotatable during the binding process. Therefore, we believe that salbutamol still has a binding activity to β_2_-AR after attachment.

These adsorbents were used to immobilize the three receptors to assess the catalytic activity using the Halo-tag® TMR ligand as a fluorescence marker. No fluorescence was observed for the control adsorbents, including bare or salbutamol/angiotensin II-coated silica gel, whereas intense florescence was observed in both salbutamol and angiotensin II-coated gel treated with the corresponding receptors (ESI Fig. 12†). This result indicated that the dehalogenase domains of the three fusion receptors retain catalytic activity even after immobilization on solid matrices.

**Fig. 5:**
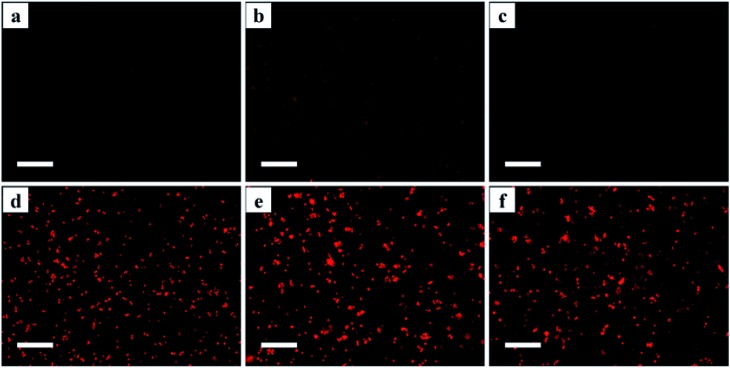
Cyanine5 maleimide-labelled silica gel coatings: (a) bare silica gel; (b) aminopropyl silica gel; (c) 6-chlorohexanoic acid-coated gel; (d) β_2_-AR-coated gel; (e) AT_1_-coated gel; (f) AT_2_-coated gel. The micrographs were obtained at a magnification of 40× using a Nikon C2 Plus confocal microscope with appropriate filter sets. Scale bar: 50 μm.

The number of cysteine residues in the gene sequences of β_2_-AR (13), AT_1_ (10) and AT_2_ (14) is much greater than that of the dehalogenase (2) incorporated into the receptors, implying that thiols are more prevalent in the receptor domains of the three Halo-tagged proteins. Cyanine5 maleimide was selected as a fluorescence marker to explore the ligand-binding activity of the Halo-tagged receptors. The control supports made of bare silica gel, aminopropyl silica gel and 6-chlorohexanoic acid-coated gel showed little fluorescence (Fig. 5a–c), but intense fluorescence was observed when the 6-chlorohexanoic acid-coated gel was treated with the three Halo-tagged receptors (Fig. 5d–f). The fluorescence distribution in each microsphere was uniform and contrasted sharply against the surrounding liquid environment. Using 6-chlorohexanoic acid-coated gel as the support, we visualized less fluorescence than the three Halo-tagged receptors when dehalogenase itself was immobilized (ESI Fig. 13†). This result suggested the uniform distribution of Halo-tagged receptors on the surface of the silica gel and confirmed covalent attachment without physical immobilization of the receptors.

### Conformation regulation by specific ligands

**Fig. 6:**
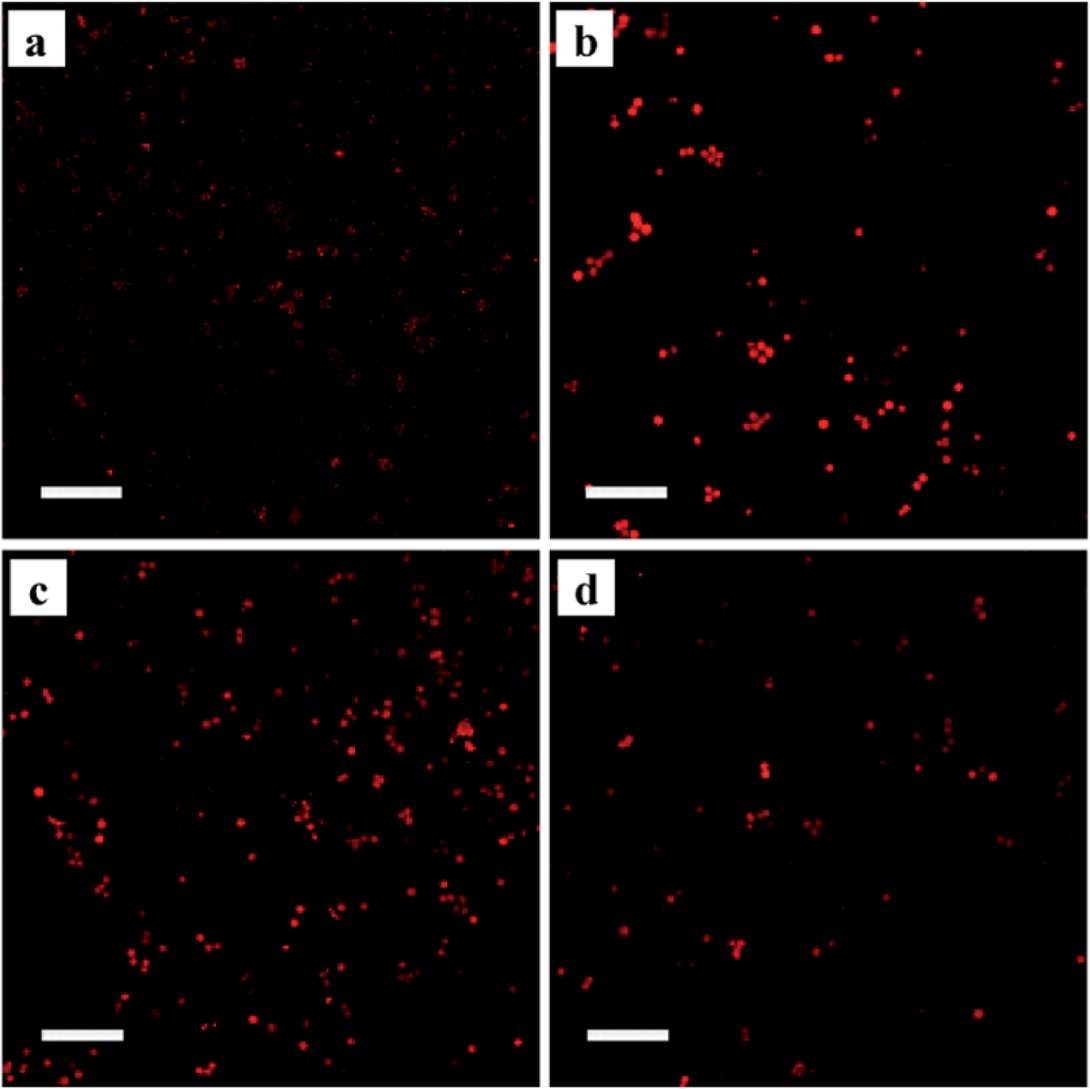
Ligand-induced conformational changes of immobilized β_2_-AR with: (a) no ligand; (b) isoproterenol; (c) salbutamol; (d) carazolol. Cyanine5 maleimide was used as a probe to label the receptor. The micrographs were obtained at a magnification of 40× using a Nikon C2 Plus confocal microscope with appropriate filter sets. Scale bar: 50 μm.

GPCRs are involved in nearly every *in vivo* physiological process *via* direct or indirect pathways. These biological responses are typically mediated through ligand-induced conformational changes, which activate heterotrimeric G proteins and lead to the generation of second messengers. Pioneering studies have proposed the classic receptor theory of active and inactive receptor conformations. The active conformation of the receptor is responsible for interacting with and activating all of the messengers. Agonists with different efficacies modulate the equilibrium between the active and inactive receptor conformations. Full agonists stabilize the active state completely, whereas partial agonists have no effect on the relative proportion of each state due to an equilibrium shift caused by stabilizing an inactive state or by occupying the orthosteric ligand-binding pocket. Recent evidence has challenged this classical view, suggesting that the ligand-induced receptor response is nonlinear. As a model GPCR, β_2_-AR has been found to signal through both G proteins and β-arrestins, which provides evidence for the existence of multiple independent signalling pathways downstream of the receptors. The possible existence of diverse receptor conformations has been investigated using MS-based quantitative analysis.^43^ Despite these insights into agonist-induced receptor conformational changes, such studies have not aided the study of immobilized GPCR conformations. To develop an approach allowing the analysis of the conformational changes in immobilized proteins, we utilized cyanine5 maleimide as a fluorescent reagent to selectively label the thiol groups of the Halo-tagged β_2_-AR, AT_1_ and AT_2_ conjugated to the surface of the silica microspheres. The ligand-specific conformations of each labelled receptor were identified using three structurally and functionally distinct ligands (ESI Fig. 14†). In opposition to the two-state conformation theory, the fluorescence intensities of Halo-tagged β_2_-AR pre-treated with a full agonist (isoproterenol), a partial agonist (salbutamol) and an antagonist (carazolol) were clearly different from each other (Fig. 6). Similar results were obtained for the Halo-tagged AT_1_ and AT_2_ when the two receptors were treated with corresponding specific ligands. These results indicate that the immobilized receptors have at least three ligand-induced conformations and confirm that receptor–ligand binding activity is nonlinear.

Here, we described a powerful approach capable of examining site-specific conformational changes in immobilized proteins. Using this methodology, we confirmed that immobilized GPCRs, including β_2_-AR, AT_1_ and AT_2_, occupy multiple conformations upon ligand stimulation. This result can profoundly inform the development of immobilized protein-based methodologies such as enzyme reactors, protein microarrays, biosensors and affinity chromatography. The basis for this effect might be the crucial role of conformational changes in bridging ligand binding affinity and therapeutic effects, thereby extending the potential application of these techniques.

### Chromatographic analysis

**Fig. 7:**
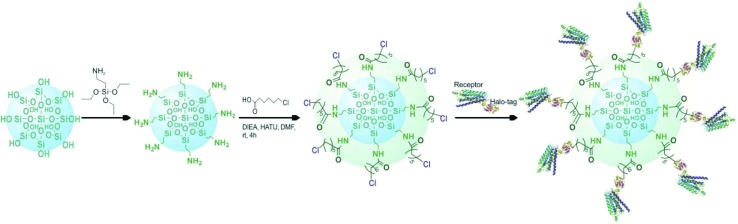
Diagram of the selective covalent capture of Halo-tagged receptors onto a chloroalkane linker-coated macroporous silica gel.

**Fig. 8:**
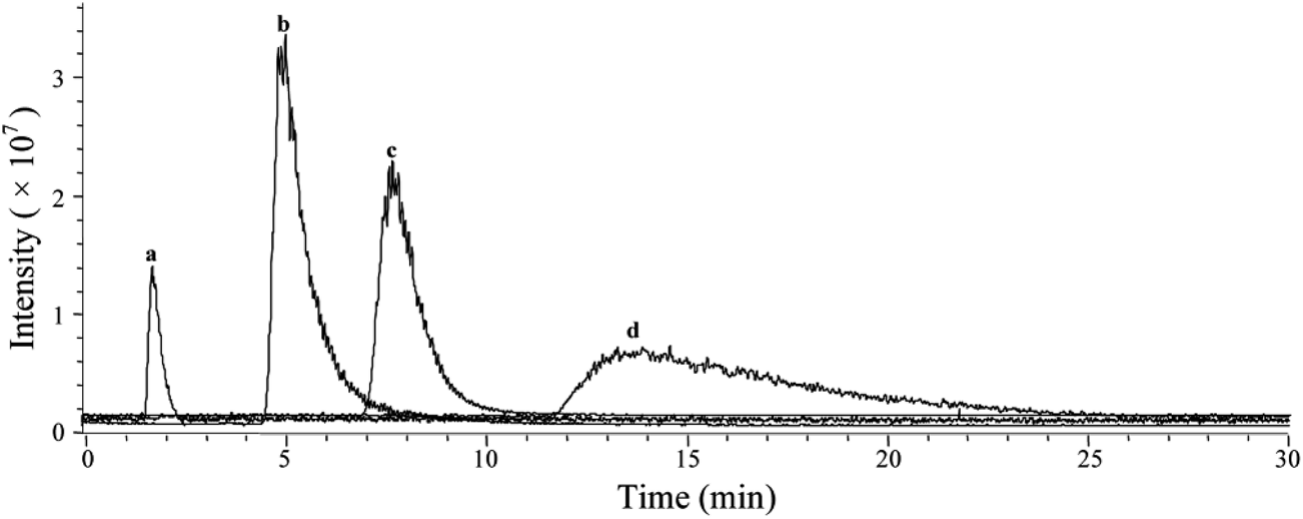
Representative chromatograms of ligands on an immobilized β_2_-AR column. Peaks are identified as: (a–d). (a) Void time determined from isoproterenol after the column was treated with 7 M guanidine hydrochloride; (b), salbutamol; (c), isoproterenol; (d), carazolol.

Based on the above mentioned characterization, we speculated that our immobilization strategy enabled higher density and improved activity of the receptors on solid support surfaces. Using β_2_-AR as a probe, we synthesized the immobilized receptor (Fig. 7) and tested this hypothesis by exploring the interaction between three drugs and the receptor with a set of chromatographic experiments. We observed diverse retention times for isoproterenol, salbutamol and carazolol on a column containing immobilized β_2_-AR (Fig. 8). The β_2_-AR antagonist carazolol showed a much longer retention time than isoproterenol (agonist) and salbutamol (partial agonist), confirming the ligand recognition specificity of immobilized β_2_-AR. Despite the asymmetry and peak tailing in the chromatographic profiles, remarkable improvements in peak width were attained compared to previous reports attaching β_2_-AR onto silica gel using a diazo reaction or the *N*,*N*′-carbonyldiimidazole method. These improvements stem from the relatively homogeneous monolayer of β_2_-AR formed using this method.

Using nonlinear chromatography, we calculated the binding parameters of the three drugs to the immobilized β_2_-AR. ESI Table 4† summarizes the capacity factors, association constants and rate constants determined in this work and collected from the literature.^44^,^45^ Compared to previous chromatographic investigations, the current immobilization methodology provided increased association constants of β_2_-AR for the three drugs. These results are expected, as our strategy has the unique advantage of minimizing the loss of protein bioactivity because isolation and purification of the protein is not necessary. These results, in particular the improved retention of protein bioactivity, have confirmed the merits of this proposed methodology.

## Experimental

### Plasmid cloning and construction

Plasmids encoding the β_2_-AR, AT_1_ and AT_2_ genes with the Halo gene at their C-terminus were constructed using BP and LR reactions in *E. coli*. DNA fragments for the β_2_-AR (Accession: NM_000024.5), AT_1_ (Accession: BC068494.1) and AT_2_ (Accession: BC095504.1) encoding attB1 and attB2 recombination sites were supplied by GeneCopoeia (Guangdong, China). To create entry clones, we mixed these DNA fragments with shuttle vectors containing attP1 and attP2 sites, followed by the addition of 5 × BP Clonase™ Reaction buffer, BP Clonase™ enzyme mix and distilled water. After a 2.0 h reaction at 25 °C, we utilized α_1_ proteinase K to block the BP Clonase™ enzyme. Two microliters of the three products were transformed into *E. coli* TOP10 cells for the selection of kanamycin-resistant entry clones. The three entry clones encoding β_2_-AR, AT_1_ and AT_2_ DNA fragments were transformed into the destination vector pReceiver-ccdB-Halo containing attR1 and attR2 sites using the LR recombination reaction to generate the expression clones pReceiver-β_2_-AR-Halo, pReceiver-AT_1_-Halo and pReceiver-AT_2_-Halo.

### Protein expression

The expression clones were transformed into *E. coli* BL21(DE3) to select positive clones with ampicillin resistance. The selected clones were cultured in LB media at 37 °C overnight and then seeded in auto-induction growth media for an additional 12.0 h incubation. The pellets were collected by centrifugation, suspended in lysis buffer and incubated for 30 min at 37 °C. The bacterial suspension was kept in an ice-bath and sonicated. The disrupted bacterial suspension was centrifuged, and the supernatant was used for the immobilization of the Halo-tagged β_2_-AR, AT_1_ and AT_2_ without further purification.

### Immobilization of fused receptors

Aminopropyl silica gel was activated with the desired linkers using an acylation reaction under the following conditions: solvent = DMF, condensing agent = 1-bis(dimethylamino)methylene-1*H*-1,2,3-triazolo[4,5-*b*]pyridinium 3-oxid hexafluorophosphate, accelerant = *N*,*N*-diisopropylethylamine, temperature = 25 °C and duration = 2.0 h. The linker-coated gel was immersed in crude lysates containing the Halo-tagged β_2_-AR, AT_1_ and AT_2_ with stirring for 1.0 h to prepare the receptor-conjugated microspheres. The physically adsorbed proteins were removed with phosphate buffer (20.0 mM, pH 7.4).

### X-ray photoelectron spectroscopy

X-ray photoelectron spectroscopy was performed in a VG ESCALAB220i-XL analyser (Thermo Scientific, Surrey, UK) at an ultrahigh vacuum surface analytical facility with a base vacuum of 1.0 × 10^–8^ Pa. Monochromatic Al Kα (1486.6 eV, 14.5 kV, 30 mA) was utilized as the incident radiation with a pass energy of 200 eV. The binding energies were calibrated with respect to the C 1s peak at 284.8 eV. A combination of a Shirley-type background and a linear-type background was used for curve fitting. All of the fittings were performed using Voigt-shaped peaks with an equal FWHM for each data set.

### Cryo-field emission scanning electron microscopy

The surface morphology for all of the samples was characterized using a Su 8010 (Hitachi, Japan) cryo-field emission scanning electron microscope equipped with a cryo-field emission gun, lower and upper secondary electron detectors and a Genesis software system. The samples were transferred under vacuum to the cryo-SEM sample preparation chamber, where the sample temperature was maintained at –150 °C by seating the sample holder on a cold stage. The samples were subsequently fractured using a cold knife to reveal a clean surface. The images were acquired at an accelerating voltage of 2 kV and a working distance of 4–5 mm.

### High resolution transmission electron microscopy

The inner microstructure of all of the samples was assessed with a FEI Tecnai G^2^ F20 S-Twin (FEI, Houston, TX, USA) transmission electron microscope equipped with a field emission gun, a scanning unit with a high angular annular dark field detector (HAADF) and an energy-dispersive X-ray spectroscope (EDAX, Tilburg, The Netherlands). The microscope was controlled by running programs, including a Tecnai user interface, a digital micrograph and Tecnai imaging and analysis. Electron micrographs were recorded either on photographic film or with a CCD camera under low-dose conditions.

### HPLC/MS/MS conditions for phospholipid identification

HPLC/MS/MS analyses of the phospholipids were performed using an Agilent 1100 LC-MSD system (Agilent Technologies, Waldbronn, Germany) consisting of a quaternary pump (G1311A), a column thermostat (G1316A), a diode-array detector (1315B), and an ion trap MS with an electrospray ionization (ESI) tandem MS interface. Data acquisitions and processing were carried out using a MSD Trap Control and Data Analysis 4.2 data system. LC separations were obtained on an Inertsil ODS-3 C-18 column (4.6 × 150 mm, 5 μm) at room temperature using ammonium acetate (5 mM) (Solvent A) and acetonitrile (Solvent B) in gradient elution at a flow rate of 0.5 ml min^–1^. The gradient elution was performed as follows: after 10 min of isocratic elution (Solvent A 50%), Solvent B was increased from 50% to 95% in 25 min along a linear gradient curve and the final elution was held for another 30 min. The injections were carried out with a Rheodyne model 7725i (Interchim, Montluçon, France) valve equipped with a 20 μl loop. The optimized ESI parameters for negative ion detection were capillary voltage set at +3.5 kV with a scan range from 100 to 1500 *m*/*z*. Nitrogen was used as the drying (10 l min^–1^, 350 °C) and nebulizing (30 psi) gas.

### Radio-ligand binding assay

Saturation binding assays were performed with [^125^I]-cyanopindolol for β_2_-AR and [^125^I]-[Sar^1^, Ile^8^]AngII for AT_1_ and AT_2_. Nonspecific binding was determined in the presence of 1 μM DL-propranolol for β_2_-AR and 1 μM unlabeled [Sar^1^, Ile^8^]AngII for AT_1_ and AT_2_. Competitive binding assays were carried out using various concentrations of ICI 118,551 (β_2_-AR selective antagonist), losartan (AT_1_ selective antagonist) and PD123319 (AT_2_ selective antagonist) as competitive agents. The specific binding was determined by subtracting the nonspecific binding from the total binding.

### Confocal microscopy analysis

Confocal microscopy analysis was performed with a Nikon C2 Plus confocal microscope (C2^+^ Nikon, Tokyo, Japan). This microscope provides 4 laser lines (405, 488, 561 and 640 nm) and is able to take images with up to 2048 × 2048 pixels resolution. The samples were placed over glass slides, covered with glass cover slips and exposed to the microscope. Images were captured and processed using the NIS ELEMENTS software provided with the instrument.

## Conclusion

This study presents a simple bioorthogonal approach for the oriented covalent immobilization of β_2_-AR, AT_1_ and AT_2_ onto microspheres as well as a comprehensive protocol to characterize the morphology and activity of immobilized receptors and validate their application in receptor chromatography for ligand–receptor interaction analysis. The immobilization strategy is fast, robust, chemoselective and has a high yield and the unique advantage of minimizing the loss of protein function. This method has the potential to become a universally applicable alternative for protein immobilization on solid supports with high efficiency, especially for proteins with low solubility. The methodology for characterizing receptor-conjugated microspheres can assess protein morphology, ligand binding activity and ligand-induced conformational changes. These characterization methods are particularly important in the development of sensitive, immobilized protein-based techniques including bioreactors, microarrays and biosensors to carefully probe biological processes. Receptor chromatography is useful for the study of ligand–receptor interactions. It is a powerful platform for rapid ligand–receptor binding analysis and drug candidate screening.

## Conflicts of interest

The authors declare no competing financial interests.

## Supplementary Material

Supplementary information
