# Supplementary material for: One-step methodology for the direct covalent capture of GPCRs from complex matrices onto solid surfaces based on the bioorthogonal reaction between haloalkane dehalogenase and chloroalkanes
Source: Chem Sci. 2017 Oct 19;9(2):446–56. doi: 10.1039/c7sc03887a (PMC5868316; doi:10.1039/c7sc03887a)
Supplement: Supplementary file 1 [file SC-009-C7SC03887A-s001.pdf]

Electronic Supplementary Material (ESI) for Chemical Science.
This journal is © The Royal Society of Chemistry 2017

Supporting Information for:

One-step methodology for the direct covalent capture of GPCRs from complex matrices onto solid surfaces based on the bioorthogonal reaction between haloalkane dehalogenase and chloroalkanes

Kaizhu Zeng[1,¶], Qian Li[1,¶], Jing Wang[1], Guowei Yin[2], Yajun Zhang[1], Chaoni Xiao[1], Taiping Fan[1,3], Xinfeng Zhao[1]*, Xiaohui Zheng[1]

[1]Key Laboratory of Resource Biology and Biotechnology in Western China, Ministry of Education, College of Life Sciences, Northwest University, Xi'an 710069, China

[2]Department of Biochemistry and Biophysics, University of North Carolina at Chapel Hill, NC, USA

[3]Department of Pharmacology, University of Cambridge, Cambridge CB2 1PD, United Kingdom

¶ Authors contribute equally

Supplementary methods

Immuno-TEM analysis

To confirm if the gleaming layer observed in classical TEM photographs was caused by Halo-tagged receptor coating, the β_2-AR-conjugated microspheres and 6-chlorocaproicacid coated gel were treated by 500 μL blocking solution (0.5% skimmed milk powder in 1×TBST, pH=7.5) for 1.0 h at room temperature. After rinsing three times with 1×TBST, the supports were suspended in 300 μL rabbit anti-human β_2-AR polyclonal antibody (Abcam, Cambridge, UK) for shaking in cold room at 4 ºC overnight. Following the removal of the residual first antibody with 1×TBST, the microspheres were suspended in 250 μL secondary antibody (gold nanoparticles labelled goat anti-rabbit IgG, Abcam, Cambridge, UK), and were incubated for 1.0 h. The result microspheres were totally rinsed with 1×TBST and dried under vacuum for TEM analysis.

Synthesis of salbutamol and angiotensin II affinity adsorbents

We synthesized salbutamol and angiotensin II affinity adsorbents with a reported N,N'- carbonyldiimidazole method.[1] Briefly, we immersed the macroporous silica gel in a MeOH/HCl solution (1:1, v/v) for 30 min. Following extra 30-min incubation in concentrated H_2SO_4, we dried the gel under a stream of nitrogen in prior to subsequent cleaning and modification to minimize cross contamination. We modified the clean gel with γ-aminopropyltriethoxysilane and activated the resultant amino-layer with N,N'-carbonyldiimidazole using acetonitrile as a solvent. The activated gel

was filtrated and rinsed with cold phosphate buffer (20 mM, pH 7.4) for the removal of residual acetonitrile. We incubated this gel with salbutamol or angiotensin II for 2.0 h under phosphate buffered environment (20 mM, pH 7.4). We used the resultant affinity adsorbents to capture the desired Halo-tagged receptor by incubating the adsorbents and the cell lysates under ice bath.

Nonlinear chromatography

We determined the association constants of isoproterenol, salbutamol and carazolol to the immobilized β_2-AR with the theory derived by Thomas and Wade.[2-3] The theory hypothesizes that the adsorption and desorption kinetics determine the level of band broadening and peak skew. Given that the axial dispersion and extra-column effects can be neglected, one can use eq. (1) to model seriously tailed and even right triangular peaks:

$$y = \frac{a_0}{a_3}\left[1 - exp\left(-\frac{a_3}{a_2}\right)\right]\left[\frac{\sqrt{\frac{a_1}{x}}I_1\left(\frac{2\sqrt{a_1 x}}{a_2}\right)exp\left(\frac{-x-a_1}{a_2}\right)}{1 - T\left(\frac{a_1}{a_2},\frac{x}{a_2}\right)\left[1 - exp\left(-\frac{a_3}{a_2}\right)\right]}\right] \quad (1)$$

$$T(u,v) = exp(-v)\int_0^u exp(-t)I_0(2\sqrt{vt})dt \quad (2)$$

where $I_0()$ and $I_1()$ are modified Bessel functions, T() is a switching function generating peak skew when the column is overloaded, a_0 is an area parameter and a_1 stands as the thermodynamic capacity factor, a_2 and a_3 are width and distortion parameters. The dissociation rate constant (k_d), association rate constant (k_a) and binding constant (K_A) can be calculated by equations: k_d=1/a_2t_0; k_a=k_dK_A; K_A=a_3/C_0.

C_0 is the concentration of the solute injected multiplied the width of the injection pulse.

All of the chromatographic experiments were performed on Agilent (Walldbronn, Germany) SL 1100 ion-trap mass spectrometer equipped with an electrospray ionization (ESI) interface, an 1100 series binary pump, a column oven, and 5.2 Chemstation software for data acquisition and processing. Chromatographic separation was achieved on a 30×4.6 mm containing immobilized β_2-AR (particle size: 7.0 μm; pore size: 300 Å). The mobile phase was acetic acid-ammonium acetate buffered solution (20 mM, pH=7.2). A post-column mobile-phase splitting valve was inserted so that only a half of the elution was diverted to electrospray ionizer. The optimum conditions for the LC–MS interface were: ion spray potential -4,500 V, nebulizer gas (N_2) pressure 35.0 p.s.i., dry gas (N_2) flow rate 8.0 L min^{-1}. The dry gas temperature was 350 °C. Multiple reaction monitoring (MRM) MS–MS was used for specific detection by measuring the characteristic ion transitions m/z 212.2 (parent ion) to m/z 194.1 (product ion) for isoproterenol, m/z 240.1 (parent ion) to m/z 166.2 (product ion) for salbutamol and m/z 299.2 (parent ion) to m/z 116.3 (product ion) for carazolol. All chromatographic experiments were performed at 25 °C with an injection volume of 5.0 μL of each drug.

The column was equilibrated with an acetic acid-ammonium acetate buffered solution (20 mM, pH=7.2) for 30.0 min. Then, 5.0 μL aqueous solutions of 40.0 μM isoproterenol, salbutamol and carazolol were independently injected to record their elution profiles. Subsequent experiments were performed by sequentially injecting a

series of concentrations of each drug onto the chromatographic system. In this case, the concentrations were 5.0-4000 (5.0, 10, 50, 500, 1000, 1500, 2000, 2500, 3000 and 4000) µM for isoproterenol, 0.5-350 (0.5, 1.0, 5.0, 10, 50, 100, 150, 200, 250 and 350) µM for salbutamol and 0.05-1000 (0.05, 0.1, 0.5, 1.0, 5.0, 10, 100, 250, 500 and 1000) µM for carazolol. At the end of each series, the column was totally equilibrated using the mobile phase (approximately 30.0 min).

1. S. C. Freitas, M. A. Barbosaand M. C. Martins, *Biomaterials,* 2010, **31**, 3772-3780.
2. H. C. Thomas, *J. Am. Chem. Soc.*, 1944, **66**, 1664-1666.
3. J. L. Wade, A. F. Bergold, and P. W. Carr, *Anal. Chem.*, 1987, **59**, 1286-1295.

Supplementary Figures

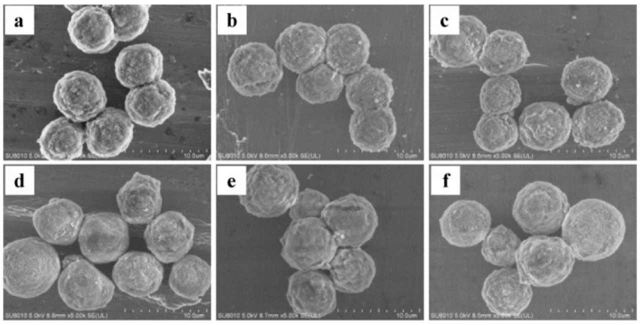

Supplementary Figure 1. Higher magnification SEM images of silica gel coatings: (a) bare silica gel, (b) aminopropyl silica gel, (c) 6-chlorohexanoic acid-coated gel, (d) β_2-AR-coated gel, (e) AT_1-coated gel, (f) AT_2-coated gel.

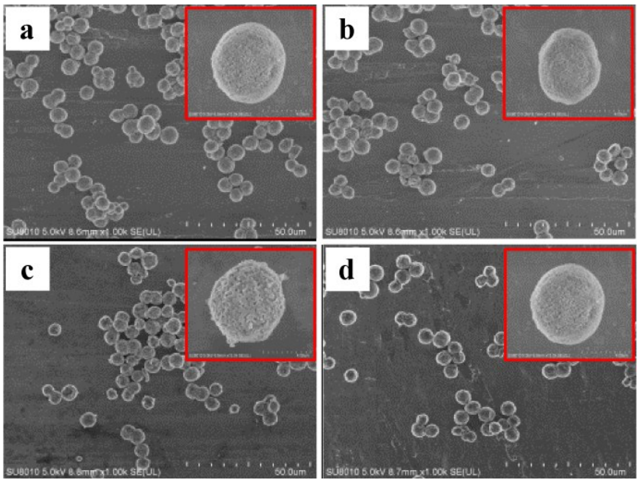

Supplementary Figure 2. SEM images of silica gel coatings by calcination at 600 °C.

(a) bare silica gel, (b) β_2-AR-coated gel, (c) AT_1-coated gel, (d) AT_2-coated gel.

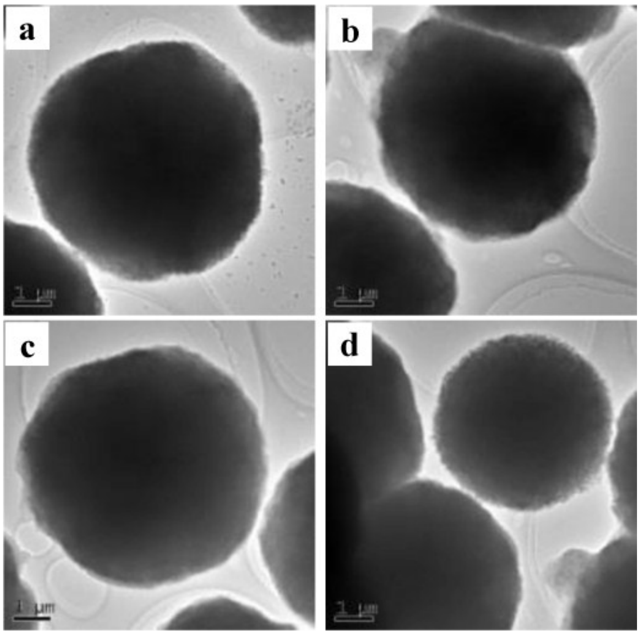

Supplementary Figure 3. TEM images of silica gel coatings: (a) bare silica gel, (b) β_2-AR-coated gel, (c) AT_1-coated gel, (d) AT_2-coated gel.

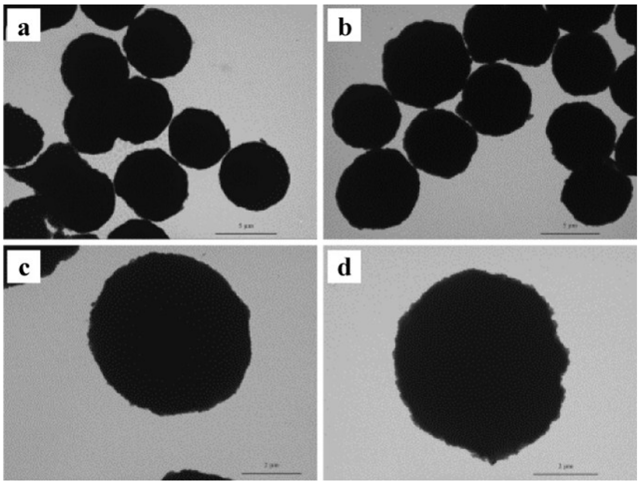

Supplementary Figure 4. Immuno-TEM images of 6-chlorohexanoic acid-coated gel (a, c) and β_2-AR-coated gel (b, d).

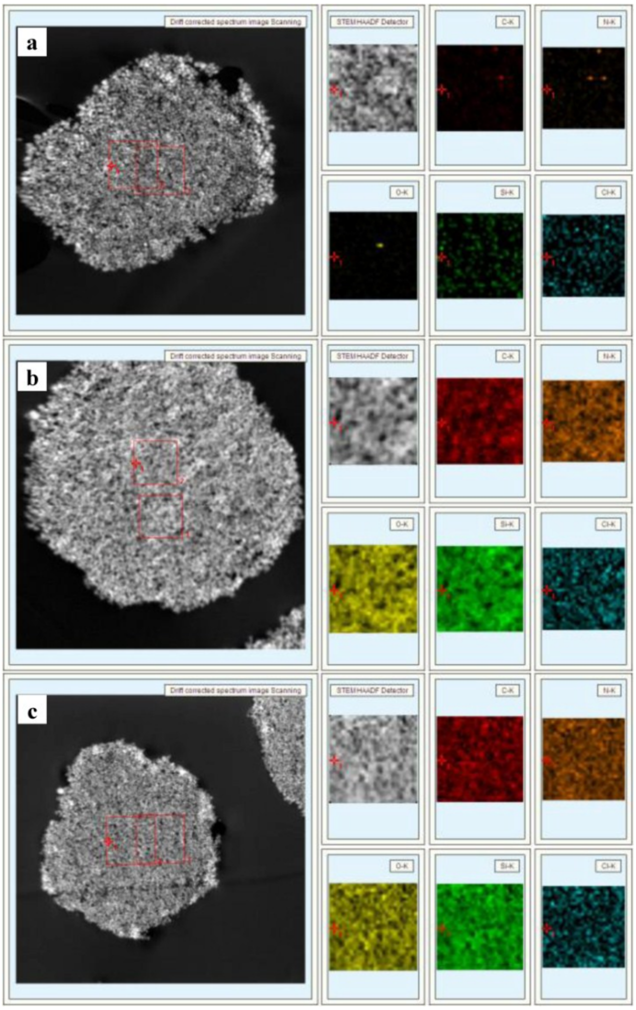

Supplementary Figure 5. STEM-HAADF images and STEM-EDS chemical maps of the cross section's middle regions of bare silica gel (a), aminopropyl silica gel (b) and 6- chlorohexanoic acid-coated gel (c).

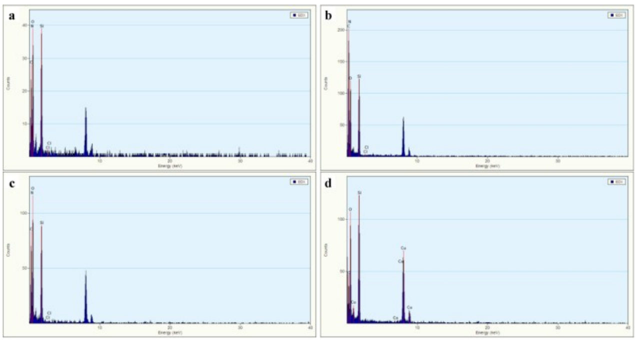

Supplementary Figure 6. EDS measurements of silica gel coatings: (a) 6-chlorohexanoic acid-coated gel, (b) β_2-AR-coated gel, (c) AT_1-coated gel, (d) AT_2-coated gel.

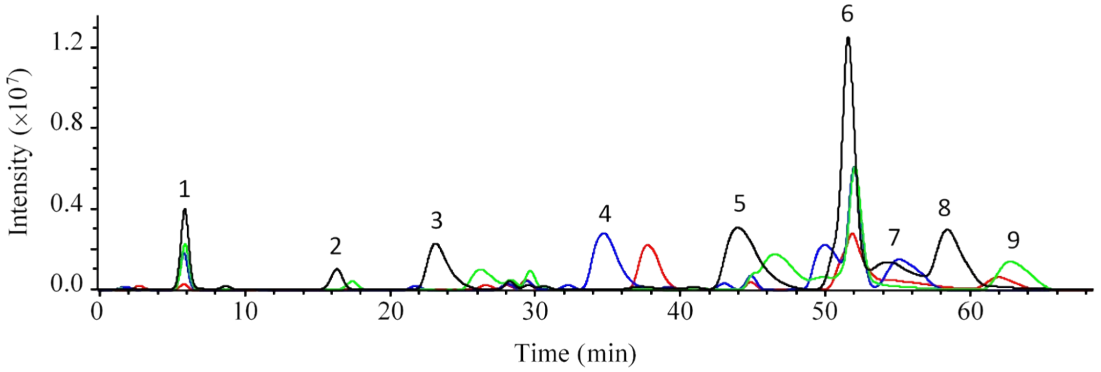

Supplementary Figure 7. HPLC/MS analysis of the phospholipids extracted from *E. coli* BL21(DE3) expressed the three receptors and the immobilized receptors. Red, *E. coli* BL21(DE3); Green, immobilized AT_1; Blue, immobilized AT_2; Black, immobilized β_2-AR. The mass patterns of peak 1-9 were summarized in supplementary table 3.

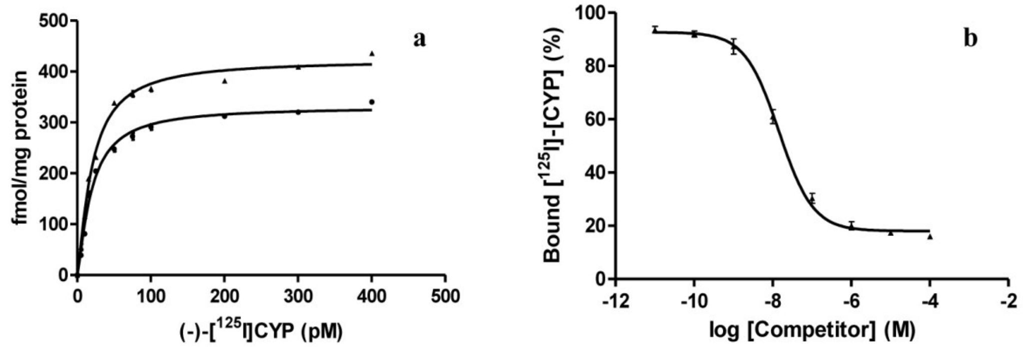

Supplementary Figure 8. Saturation (a) and competition (b) binding for (-)-[^{125}I]CYP to β_2-AR. In (a), the binding data of saturation investigation have been fitted to a one-site hyperbolic binding curve by nonlinear regression analysis. (●), membranes prepared from *E. coli* BL21(DE3) expressed β_2-AR; (▲), immobilized β_2-AR. In (b), the binding data have been fitted to a one-site competition curve for the specific antagonists ICI 118,551.

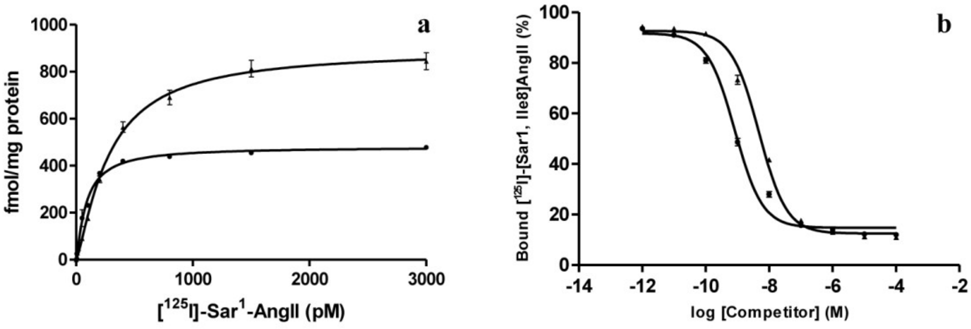

Supplementary Figure 9. Saturation (a) and competition (b) binding for $[^{125}I]$-Sar^1-AngII to immobilized AT_1 and AT_2. In (a), (▲) represented immobilized AT_1; (●) was the data for immobilized AT_2. In (b), the competition binding experiment, the binding data have been fitted to a one-site competition curve for the specific antagonists (▲, losartan; ●, PD123319).

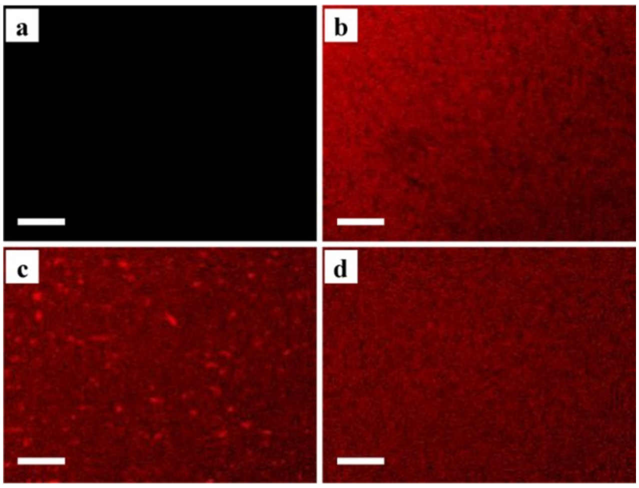

Supplementary Figure 10. HaloTag® ligand labeling of uninduced *E. coli* (a) and *E. coli* expressing β_2-AR (b), AT_1 (c) and AT_2 (d). The morphology was recorded by Nikon C2 Plus confocal microscope ($C2^+$ Nikon, Tokyo, Japan). Scale bar: 50 μm.

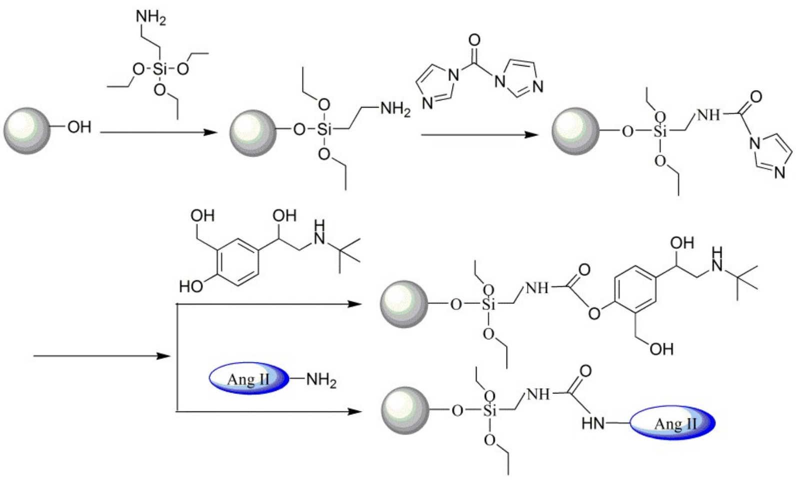

Supplementary Figure 11. Diagram for attaching salbutamol and angiotensin II onto the surface of silica gel.

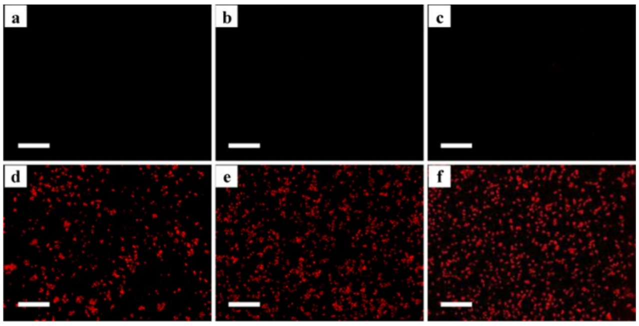

Supplementary Figure 12. HaloTag® ligand labeling of silica gel coatings: (a) salbutamol-coated silica gel, (b) angiotensin II-coated silica gel, (c) angiotensin II-coated silica gel, (d) β_2-AR-coated gel, (e) AT_1-coated gel, (f) AT_2-coated gel. Scale bar: 50 μm.

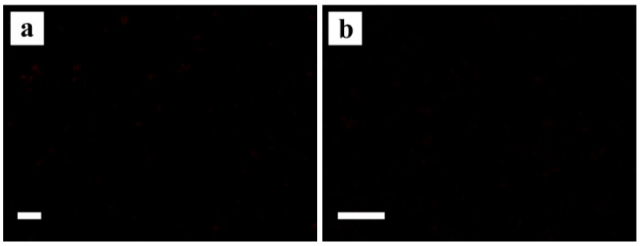

Supplementary Figure 13. Cyanine5 maleimide labeling of silica gel coatings dehalogenase using linker 3 at magnifications of 20× (a) and 40× (b). Scale bar: 50 μm.

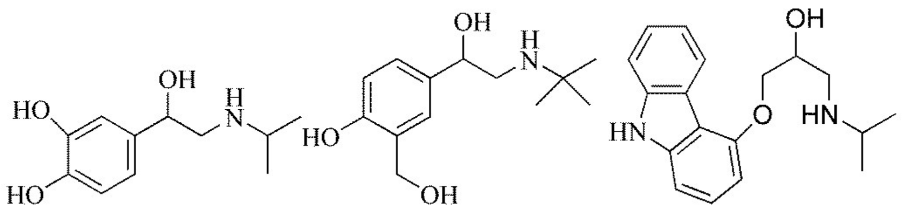

a **b** **c**

Supplementary Figure 14. Chemical structures of β_2-AR ligands: (a) isoproterenol; (b) salbutamol; (c) carazolol

Supplementary Table 1 Structures of the linkers used for immobilization of Halo-tagged receptors

Number	Structure	Name	XlogP[a]	Bound receptor (μg/g)
1	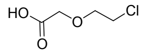	2-(2-chloroethoxy)acetic acid	-0.19	210±6.3
2	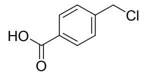	4-(chloromethyl)benzoic acid	2.5	295±8.6
3	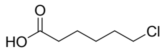	6-chlorohexanoic acid	0.9	347.2±10.4
4	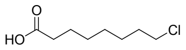	8-chlorooctanoic acid	1.2	310.7±11.7
5	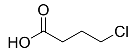	4-chlorobutanoic acid	0.6	163.1±7.5

[a] The data were collected from PubChem compound database: https://pubchem.ncbi.nlm.nih.gov/

Supplementary Table 2 The characteristic C 1s signal positions of free and immobilized receptors observed in this work (eV)

Bonds	β_2-AR	Immobilized β_2-AR	AT_1	Immobilized AT_1	AT_2	Immobilized AT_2
C−C	284.92±0.04	284.89±0.02	284.93±0.04	284.87±0.01	284.91±0.03	284.90±0.06
C−N	286.14±0.04	286.20±0.06	286.16±0.04	286.32±0.07	286.18±0.05	286.09±0.04
C−O	286.93±0.03	286.88±0.06	286.95±0.02	286.90±0.03	287.01±0.08	286.86±0.08
O=C−N	288.30±0.10	288.34±0.08	288.28±0.04	288.24±0.04	288.36±0.12	288.41±0.06
O=C−OH	288.94±0.03	288.90±0.02	288.95±0.06	288.86±0.10	288.97±0.08	288.91±0.03
C-N_3	289.23±0.12	289.30±0.06	289.21±0.10	289.19±0.08	289.25±0.07	289.24±0.08

Supplementary Table 3 Identification of phospholipids (PLs) specifically bound to the immobilized receptors; product ions observed in negative ESI MS/MS.

Peak	t_R (min)	$[M-H]^-$	Phospholipids	RCH_2 CO_2-sn-1	RCH_2 CO_2-sn-2
1	5.9	743.9	PG C18:1/C16:1	281.1	253.3
2	16.8	703.8	PE C15:0/C16:1	227.2	266.9
3	23.2	720.2	PG C16: 0/C16:0	254.8	255.2
4	35.0	746.3	PG C16:0/C18:1	255.0	281.3
5	44.2	688.1	PE C16:0/C16:1	240.7	268.0
6	51.7	796.2	PG C18:1/C18:1	281.0	281.2
7	54.3	689.4	PE C16:0/C16:0	255.3	255.1
8	58.6	715.2	PE C16:0/C18:1	255.2	281.3
9	63.0	741.3	PE C 18:1/C 18:1	281.3	280.6

Supplementary Table 4 Binding parameters of specific ligands to immobilized β_2-AR by nonlinear chromatography

Ligands	Capacity factors	Association constants (L/mol)		Rate constants (k_d) (/s)
		This work	Literature	This work
Isoproterenol	5.11±0.02	$(6.87\pm0.85)\times10^{6}$	[a] $(4.37\pm0.91)\times10^{6}$	0.056±0.002
	4.79±0.03			
	4.65±0.05			
	4.12±0.02			
	3.76±0.01			
	3.25±0.04			
	2.76±0.05			
	2.45±0.03			
	2.21±0.01			
	2.09±0.04			
Salbutamol	2.98±0.01	$(1.14\pm0.05)\times10^{6}$	[a] $(1.02\pm0.07)\times10^{6}$	1.40±0.07
	2.83±0.02		[b] $(5.84\pm0.05)\times10^{3}$	
	2.64±0.03			
	2.51±0.02			
	2.40±0.03			
	2.23±0.01			
	2.07±0.01			
	1.96±0.02			
	1.75±0.01			
	1.63±0.03			
Carazolol	9.42±0.12	$(2.82\pm0.36)\times10^{8}$	[a] $(3.09\pm0.50)\times10^{10}$	0.16±0.02
	8.64±0.06			
	8.17±0.04			
	7.40±0.08			
	7.00±0.07			
	6.63±0.11			
	6.27±0.03			
	5.98±0.06			
	5.60±0.09			
	5.37±0.05			

Notes:

[a] Data was collected from references [44] where association constant was determined with radio-ligand binding assay

[b] Data was collected from references [45] where association constant was determined with zonal elution chromatographic method
